# Supplementary material for: A unified framework for species spatial patterns: Linking the occupancy area curve, Taylor's Law, the neighborhood density function and two‐plot species turnover
Source: Ecol Lett. 2021 Aug 4;24(10):2043–53. doi: 10.1111/ele.13788 (PMC8518128; doi:10.1111/ele.13788)
Supplement: Supplementary file 4 — Supplementary Material [file ELE-24-2043-s004.zip › calcs/2-sim-thomas/thomas-plots/_108_50.pdf]

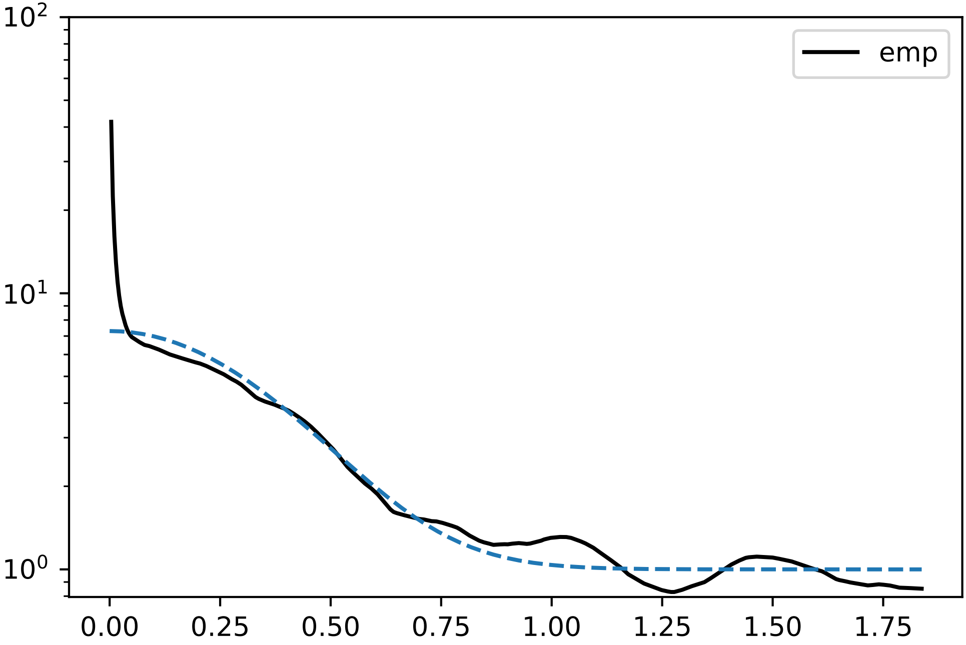

emp
10^2
10^1
10^0
0.00
0.25
0.50
0.75
1.00
1.25
1.50
1.75
